# ST-Elevation Myocardial Infarction Precipitated by Disseminated Intravascular Coagulation: A Therapeutic Challenge

**DOI:** 10.7759/cureus.37119

**Published:** 2023-04-04

**Authors:** Wahab J Khan, Dawlat Khan, Anum Nadeem, Abdul Wadood, Ifrah Nadeem, Muhammad Asif

**Affiliations:** 1 Internal Medicine, University of South Dakota Sanford School of Medicine, Sioux Falls, USA; 2 Physiology, Rashid Latif Medical College, Lahore, PAK; 3 Internal Medicine, National Hospital and Medical Center, Lahore, PAK

**Keywords:** coronary artery thrombosis, post-splenectomy, ste-acs, st-elevation myocardial infarction (stemi), disseminated intravascular coagulation (dic)

## Abstract

Acute coronary syndrome (ACS) can manifest as ST-elevation myocardial infarction (STEMI), non-ST-elevation myocardial infarction (NSTEMI), and unstable angina (UA). Common etiologies for STEMI include atherosclerotic plaque disruption or erosion manifesting as type 1 myocardial ischemia (MI). Causes of type 2 MI presenting as STEMI may include spontaneous coronary artery dissection, coronary artery spasm, and coronary embolism. STEMI is an emergency mandating immediate coronary intervention. We present a case of STEMI as a complication of disseminated intravascular coagulation (DIC). This case highlights the unique challenge of managing STEMI with active DIC.

## Introduction

Acute coronary syndrome (ACS) is a type of coronary heart disease that refers to three common entities; ST-elevation myocardial infarction (STEMI), non-ST-elevation myocardial infarction (NSTEMI), and unstable angina (UA) [1]. The basic theme behind ACS is decreased blood flow to the heart muscle which could be due to various causes. The most common mechanisms are atherosclerotic plaque disruption leading to plaque rupture or erosion and instant thrombosis with complete or near complete occlusion. Other mechanisms include coronary dissection, vasospasm, and coronary embolism, usually associated with left ventricular thrombus [2]. Herein we report a case of STEMI with disseminated intravascular coagulation (DIC) in a young patient without traditional cardiac risk factors or a family history of significant coronary artery disease.

## Case presentation

A 38-year-old male with a medical history of hereditary spherocytosis status post splenectomy 20 years ago presented to ER for a 1-day duration of fever of up to 103^o^F, nausea, vomiting, and chills. He also complained of easy bruising and had a purplish rash on his nose and cheeks. He was not taking any prescription or over-the-counter medications. Initial vital signs included a heart rate of 115 bpm, respiratory rate (RR) of 32/min, oxygen saturation (SaO_2_) of 96%, BP of 90/55 mmHg, and a temperature of 98^o^F. Physical examination was consistent with an acutely ill young patient with petechial rash and purpura on his face and extremities. HR was regular, and lungs were clear to auscultation. Labs upon presentation are given below (table 1).

**Table 1:** Laboratory values at the time of admission WBC: White blood cells; Hb: Hemoglobin; BUN: Blood urea nitrogen; CO_2_:Serum total carbon dioxide; AST: Aspartate aminotransferase; ALT: Alanine aminotransferase; ALP: Alkaline phosphatase; INR: International normalized ratio; APTT: Activated partial thromboplastin time

Lab	Value	Reference value
WBC	28.6 k/uL	4-11 k/uL
Hb	12.7 g/dL	13.5-16.9 g/dL
Platelets	44 k/uL	150-400 k/uL
Sodium	139 mmol/L	135 - 145 mmol/L
Potassium	4.7 mmol/L	3.5 - 5.1 mmol/L
Creatinine	4.15 mg/dL	0.7 - 1.3 mg/dL
BUN	39 mg/dL	7-25 mg/dL
CO2	14 mmol/L	21-31 mmol/L
Albumin	3.1 g/dL	3.5-5.7 g/dL
Total bilirubin	2.8 mg/dL	0.3-1.0 mg/dL
AST	388 U/L	13-39 U/L
ALT	263 U/L	5-25 U/L
ALP	81 U/L	34-104 U/L
CRP	370 mg/dL	< 0.5 mg/dL
Procalcitonin	309 ng/mL	<0.07 ng/mL
Lactate	10.1 mmol/L	0.5-2.0 mmol/L
INR	2.7	<1.1
APTT	89 Sec	24-36 Sec
Fibrinogen	156 mg/dL	148-535 mg/dL
D-Dimer	>=20.00 ug/mL FEU	< 0.5 ug/mL FEU

**Figure 1:**
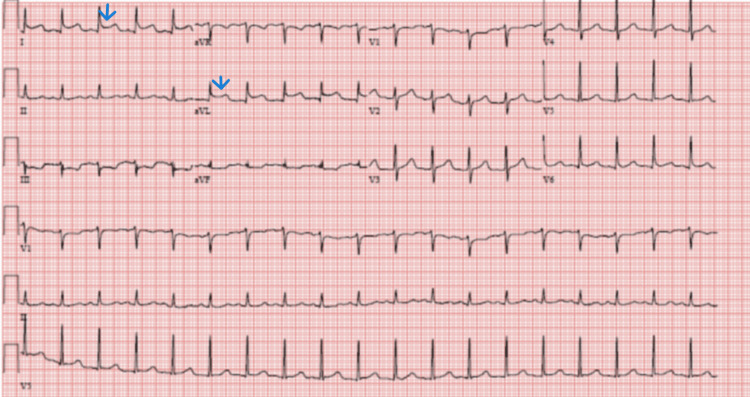
Initial EKG showing acute ST-segment elevation in lateral leads.

**Figure 2:**
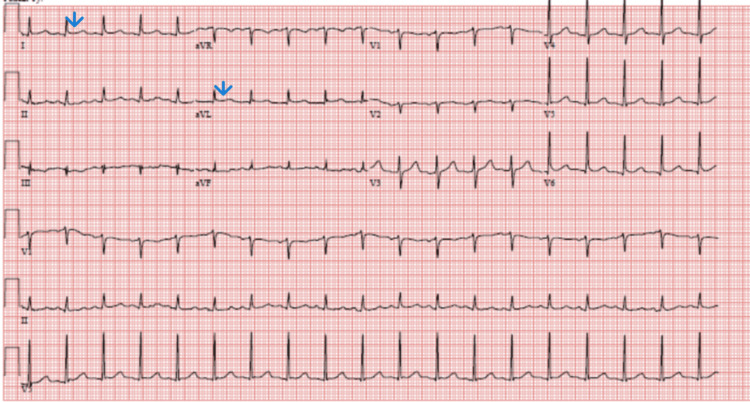
Subsequent EKG showing subsiding ST segment elevation in lateral leads.

**Figure 3:**
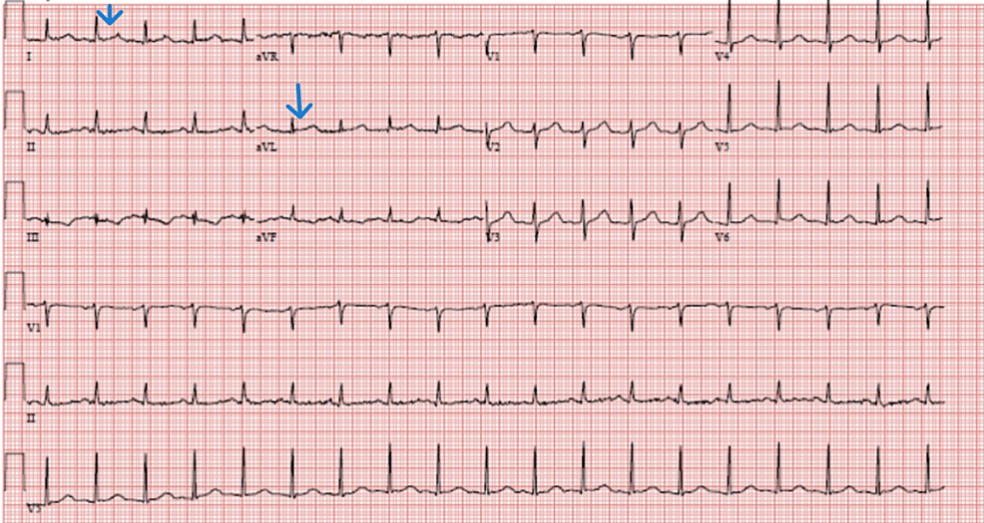
EKG showing complete resolution of ST segment elevation in lateral leads

He was admitted to the ICU for septic shock and DIC management. Hemodynamics improved with minimal vasopressor support, but the course was complicated by traumatic hematuria due to a urinary catheter that was managed conservatively. On hospital day one, he developed sudden onset precordial pressure-type chest pain and dyspnea. A stat EKG showed ST elevation involving lateral leads (fig 1). STEMI was diagnosed but could not be taken to the cardiac catheterization lab due to concurrent thrombocytopenia, AKI, DIC, ongoing hematuria, and septic shock. Aspirin and clopidogrel were loaded along with initiating a heparin drip. However, the heparin drip had to be stopped within a few hours due to worsening hematuria and new-onset epistaxis. Clopidogrel was also discontinued, and the patient was managed with aspirin alone. Troponin I level increased to 7.929 (n< 0.03) with a peak of 27.040 (table 2). Echocardiogram showed an ejection fraction of 55-60 % without valvular abnormalities or evidence of patent foramen ovale. A duplex of all four extremities showed non-occlusive right common femoral deep venous thrombosis. Fortunately, chest pain resolved with medical management, and EKG changes were reversed within 24 hours (figures 2, 3). Most of the labs, including blood cultures and serologies for pneumonia, hepatitis viruses, HIV, Q fever, Lyme, Babesia, and Ehrlichia, were negative except for the finding of enteropathogenic E.coli in the stools. The patient developed renal dysfunction requiring transient dialysis. He improved with broad-spectrum antibiotics.

**Table 2:** Troponin I trend showing the acute rise and fall concordant with EKG changes.

Time	Troponin I value in ng/ml (n <0.03 ng/ml)
At admission	0.173
At the onset of chest pain	7.929
4 hours from chest pain	22.6
10 hours from chest pain	27.04
16 hours of chest pain	19.17

Over 7 days, his hemodynamic parameters, lab values, and renal function improved to normal. He was discharged in stable condition to an acute rehabilitation facility with guideline-directed medical therapy. Cardiac catheterization was discussed with the patient. He elected not to proceed with angiography due to the complete reversal of symptoms and EKG findings with treating the cause, i.e., DIC and infection. He remains asymptomatic and is back to his baseline functional status with no limitations at three months follow-up.

## Discussion

Myocardial infarction (MI) is defined as acute myocardial injury detected by abnormal cardiac biomarkers in the setting of evidence of acute myocardial ischemia [3]. Myocardial ischemia may manifest as EKG changes in combination with various typical anginal symptoms such as precordial, jaw, upper extremities, or neck pain, or it may appear as angina equivalents such as dyspnea, fatigue, and gastrointestinal symptoms. Other presentations include cardiac arrest and arrhythmia [4].

On the other hand, the acute myocardial injury itself may be ischemic or non-ischemic and is defined by a rising or falling pattern of cardiac troponins. Ischemic causes include coronary artery disease, e.g., atherosclerotic plaque disruption with thrombosis or coronary embolism, coronary vasospasm or dissection, and demand/supply issues such as severe anemia, hypertensive crisis, hypoxia, and shock. Non-ischemic examples include cardiomyopathies, myocarditis, cardiac contusion, and viral infections [5,6].

Based on treatment strategies, MI has been traditionally classified into STEMI, NSTEMI, and UA. However, MI may be classified differently based on pathologic etiologies and clinical settings. Most recently, it has been classified into five types (type I through type V) and other unclassified types [3]. Type I is related to atherosclerotic plaque rupture or erosion, while type II is usually associated with supply and demand issues. Type III is sudden cardiac death suspected to occur due to MI and not due to non-ischemic myocardial injury but could not be confirmed antemortem. Other types in this classification include class IV (PCI-related) and class V (CABG related). In addition to this classification, several other types have been described in the literature, like re-infarction, recurrent infarction, post-operative MI, and MI due to non-obstructive coronary arteries (MINOCA) [3]. 

It is imperative to note that STEMI may occur in types I and II MI. Studies have shown a 3% to 24% incidence of ST elevation in patients diagnosed with type II MI [7]. 

A few causes of STEMI within the type II MI category may include spontaneous coronary artery dissection, coronary artery spasm, and coronary embolism or potentially thrombosis from a deranged coagulation cascade, as happens in DIC [2,8]. The criteria to diagnose type II MI requires the detection of a rise or fall of cardiac troponin values with at least one value above the 99th percentile and at least one other finding from the following; 1) symptoms of acute myocardial ischemia; 2) new ischemic ECG changes; 3)Development of pathological Q waves; 4)Imaging evidence of new loss of viable myocardium or new regional wall motion abnormality in a pattern consistent with an ischemic etiology [3].

Treatment should focus on acuity and etiology and follow standard ACS guidelines regardless of the classification. It may include blood transfusion, volume management, blood pressure and heart rate control, and coronary evaluation. However, It may not be possible in all the cases, such as in our patients, creating a therapeutic challenge.

Our patient had STEMI with likely type II MI. According to the definition, he had typical ischemia symptoms of mid-epigastric chest pain and EKG findings with troponin elevation. It is unclear whether he had in-situ coronary thrombosis related to DIC or was due to coronary embolism in the background of DIC and thrombosis elsewhere. Another explanation could be vasospasm leading to these findings. Nonetheless, his case presents a unique challenge with no clear guidelines to steer through this situation. Primarily such cases are managed medically, and decisions are made according to the clinical situation [9,10]. Fortunately, EKG findings reversed within 24 hours, indicating the potential clot dissolution. He has returned to baseline activity status without angina or functional limitations, and his renal function has recovered completely.

## Conclusions

DIC, as the name implies, can lead to coagulation or thrombosis, and it can involve the coronary arteries manifesting as STEMI. Although these cases of ACS with DIC should be approached according to standard guidelines, it poses a unique challenge with a simultaneous bleeding tendency and thrombocytopenia, making traditional therapies, including anticoagulation and dual antiplatelet use, risky or contraindicated. However, this case of treating such patients with 81 mg of aspirin alone is a good learning experience. More data about the outcomes of such patients is needed to devise a safe treatment strategy in the future.
